# Plant mitochondrial retrograde signaling: post-translational modifications enter the stage

**DOI:** 10.3389/fpls.2012.00253

**Published:** 2012-11-12

**Authors:** Markus Hartl, Iris Finkemeier

**Affiliations:** Department Biology I, Ludwig Maximilians University MunichPlanegg-Martinsried, Germany

**Keywords:** plants, mitochondria, retrograde signaling, post-translational modifications, metabolites

## Abstract

Beside their central function in respiration plant mitochondria play important roles in diverse processes such as redox homeostasis, provision of precursor molecules for essential biosynthetic pathways, and programmed cell death. These different functions require the organelle to communicate with the rest of the cell by perceiving, transducing, and emitting signals. As the vast majority of mitochondrial proteins are encoded in the nuclear genome, changes in mitochondrial status must be fed back to the nucleus to coordinate gene expression accordingly, a process termed retrograde signaling. However, the nature of these signaling pathways in plants and their underlying signaling molecules – or indirect metabolite or redox signals – are not completely resolved. We explore the potential of different post-translational modifications (PTMs) to contribute to mitochondrial retrograde signaling. Remarkably, the substrates used for modifying proteins in many major PTMs are either central metabolites or redox-active compounds, as for example ATP, acetyl-CoA, NAD^+^, and glutathione. This suggests that the metabolic status of organelles and of the cell in general could be indirectly gaged by the enzymes catalyzing the various PTMs. We examine the evidence supporting this hypothesis with regard to three major PTMs, namely phosphorylation, lysine acetylation, and glutathionylation and assess their potential to regulate not only organellar processes by modifying metabolic enzymes but also to influence nuclear gene expression.

## INTRODUCTION

**FIGURE 1:**
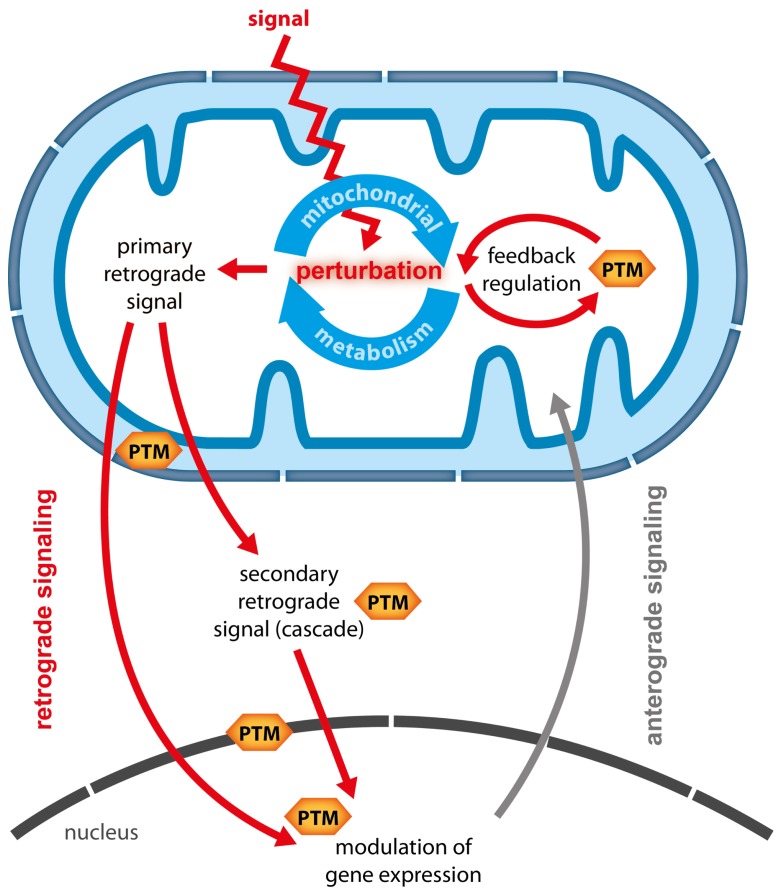
**Potential functions of PTMs in mitochondrial retrograde signaling.** When an external signal or stress causes perturbations in mitochondrial metabolism several responses are possible and PTMs could contribute on multiple levels. (1) Mitochondrial metabolism is adapted via feedback regulation, during which PTM catalyzing enzymes could alter enzymatic activity in response to changes in metabolic status. (2) Metabolic intermediates are generated as primary retrograde signals, which either travel directly to the nucleus, or which are perceived at the mitochondrial boundary or in the cytosol and then translated into secondary signals or signaling cascades. PTMs are likely to be involved in the generation and transduction of such secondary signals. Moreover, PTMs could transduce signals across the inner mitochondrial or the nuclear membrane. In the nucleus PTMs of histones, transcription factors or transcriptional complexes could translate mitochondria-derived signals into specific changes in gene expression.

Plant mitochondria are central hubs in the conversion of energy and redox homeostasis and are connected to metabolic pathways from different subcellular compartments. Hence, mitochondria are ideally placed to act as sensors of the energetic and metabolic status of the plant cell (Sweetlove et al., 2007; Millar et al., 2011). Perturbations of the cellular energy status can lead to a reconfiguration of mitochondrial activities which in turn have profound effects on other cellular compartments, including major changes in nuclear gene expression (NGE) and photosynthetic activity (Rhoads, 2011; Schwarzländer et al., 2012). Changes in NGE which are triggered by signals derived from metabolic perturbations in organelles are termed retrograde responses (RRs) and are considered important means of tuning anterograde regulation to retain metabolic plasticity (Rhoads, 2011). However, so far the well understood RRs were uncovered mainly in yeast and animals, whereas in plants conclusive evidence for the exact nature of particular retrograde signals and their transduction is largely lacking. This holds especially true for mitochondrial RR (MRR) for which so far only putative signaling candidates and one downstream transcription factor (ABI4) could be identified (Gray et al., 2004; Giraud et al., 2009). The difficulties in elucidating the nature of RR might originate in their possible complexity. Recently, Leister (2012) defined characteristics of retrograde signals and theoretically explored different scenarios of how single and multiple retrograde signals could transduce information to the nucleus in an isolated or coordinated fashion. In general two types of retrograde signals can be distinguished (**Figure 1**):

1. Primary or direct retrograde signals are generated in the organelle and either passively diffuse or are actively transported to the nucleus, where they modulate NGE. Primary retrograde signals are therefore most likely metabolic intermediates generated from metabolic pathways in the respective organelles.
2. Secondary or indirect signals are activated by primary signals inside or outside the organelle, and travel to the nucleus or activate a cascade of signaling events that eventually alter NGE.

Post-translational modifications (PTMs) are ideally suited for signal integration and thus present a likely mechanism to transduce indirect retrograde signals. PTMs such as phosphorylation have key roles in signal transduction and amplification of cytosolic signaling cascades. It is therefore conceivable that primary mitochondrial signals could generate secondary signals (Møller and Sweetlove, 2010) or activate signal transduction cascades that traverse the cytosol. Here, we discuss evidence that PTMs connected to mitochondrial energy and redox metabolism occurring both outside and inside mitochondria are potential key players in transducing indirect MRR signals. We propose that recent technological advances in high resolution mass-spectrometry and sample preparation will allow wide and largely unbiased screens for mitochondrial PTMs and their dynamics (Witze et al., 2007; Choudhary and Mann, 2010). Such PTM-based analyses could help to elucidate the black box of MRR and to reveal some of its signaling components.

## POST-TRANSLATIONAL MODIFICATIONS – LIGHT IN THE BLACK BOX OF MITOCHONDRIAL SIGNALING?

Post-translational modifications provide a powerful mechanism to rapidly and temporarily alter protein functions and locations in the cell, and they are also capable of providing information to regulatory proteins by creating docking sites for PTM-recognition domains (Deribe et al., 2010). PTMs which are dynamic, rapid, and reversible are suitable to direct transient changes in cellular metabolism and signaling. Intriguingly, the substrates of several major PTM-modifying enzymes are either central metabolites or redox-active compounds, as for example ATP, acetyl-CoA, NAD^+^, and glutathione, which suggests that the activity of these enzymes could be directly coupled to the energy status or redox status of the organelle.

## PHOSPHORYLATION

Although a few phosphorylation-regulated mitochondrial enzymes are well established, such as the pyruvate dehydrogenase (Gemel and Randall, 1992), only recently the regulatory role of phosphorylation in mitochondrial signal processing and metabolic regulation has begun to emerge (Pagliarini and Dixon, 2006; Ito et al., 2007; Juszczuk et al., 2007; Foster et al., 2009). There are indications that all major kinase signaling pathways in mammals can target the mitochondrion and influence mitochondrial function (Horbinski and Chu, 2005; Pagliarini and Dixon, 2006). Recent publications describe 77 phosphoproteins in mitochondria from human muscle cells and 181 phosphoproteins from murine cardiac mitochondria (Deng et al., 2011; Zhao et al., 2011). However, despite remarkable efforts merely 22 phosphoproteins are reported for *Arabidopsis* (Ito et al., 2009) and 14, mostly different, phosphoproteins for potato (Bykova et al., 2003). It is very likely that mitochondrial status and signaling varies with tissue and environmental conditions, which could explain why putative phosphoproteins remained undetected in studies concentrating on cell cultures or particular tissues. Similar to the fragmentary mitochondrial phosphoproteome, the available data describing mitochondrial protein kinases and phosphatases in plants is scarce (Heazlewood et al., 2004; Juszczuk et al., 2007). Several kinases and phosphatases attached to the outer mitochondrial membrane have recently been identified, but their possible role in mitochondrial signaling remains to be explored (Duncan et al., 2011; Sun et al., 2012). A large-scale yeast two-hybrid screen for interactors of mitogen-activated protein kinases (MAPKs) in rice indicates that these kinases could phosphorylate mitochondrial proteins (Singh et al., 2012). Lundquist et al. (2012) suggested that the so far largely neglected ABC1K protein kinase family could further fill the gap of mitochondrial kinases but clearly more experimental work will be necessary.

The points mentioned above indicate that phosphorylation might influence mitochondrial function but whether it could also influence MRR remains an open question. First evidence that plant MRR is indeed partially regulated via phosphorylation comes from the observation that the citrate-dependent induction of the mitochondrial alternative oxidase can be inhibited by the protein kinase inhibitor staurosporine (Djajanegara et al., 2002). Furthermore, Takahashi et al. (2003) demonstrated that spermine-induced functional changes in tobacco mitochondria trigger a MAPK-cascade and activate specific hypersensitive response genes. However, both pathways were not further explored yet.

Mitochondrial signals could activate phosphorylation-dependent signaling cascades inside or outside the organelle, which transduce MRR. Strong support for the existence of such a mechanism was found in yeast, where the “RTG-dependent pathway” represents one of the best-studied MRR. It has been shown that perturbations of mitochondrial functions in yeast result in lowered mitochondrial membrane potentials, eliciting a kinase-based response that regulates the phosphorylation status and translocation of specific TFs to the nucleus (Liu and Butow, 2006; Jazwinski, 2012). Candidates for phosphorylation-dependent signaling involved in MRR in plants are SNF1-related kinases (SnRKs), with the cytosolic SnRK1 in particular, and the target of rapamycin (TOR) protein kinases. SnRK1 and TOR belong to different evolutionary conserved families of protein kinases, which likely function as master regulators and sensors of energy and nutrient metabolism during growth and development as well as under energy-depleting stress conditions (Polge and Thomas, 2007; Baena-González and Sheen, 2008; Dobrenel et al., 2011; Ghillebert et al., 2011; Ren et al., 2011; Xiong and Sheen, 2012). Although some important modulators of SnRK1- and TOR-activity have been described the main metabolic signals that activate these kinases remain unknown (Baena-González and Sheen, 2008; Ghillebert et al., 2011; Xiong and Sheen, 2012). Interestingly, there is an overlap in regulated transcripts between SnRK1-mediated responses and specific conditions of mitochondrial dysfunction (Schwarzländer et al., 2012) and it will be interesting to ascertain whether SnRK1 integrates metabolic signals from mitochondria, thus contributing to MRR. In yeast and mammals TOR signaling is vital for the maintenance of mitochondrial respiration and it is involved in the MRR (Schieke and Finkel, 2006). For example mammalian TOR (mTOR) interacts with PGC1α (Cunningham et al., 2007), a transcriptional activator regulating mitochondrial biogenesis and activity. Recent results in *Arabidopsis* suggest that TOR could have a similar function in plants but the exact mechanisms remain to be discovered (Leiber et al., 2010; Xiong and Sheen, 2012).

## LYSINE ACETYLATION

Acetylation of the ε-amino group of lysine residues on proteins has recently emerged as a major PTM of proteins that has the potential to rival the regulatory role of phosphorylation (Choudhary et al., 2009; Norvell and McMahon, 2010). For a long time the research on lysine acetylation of proteins has concentrated almost exclusively on histones and transcriptional regulation. However, lysine acetylation of non-histone proteins was confirmed to be a widespread PTM in prokaryotes and eukaryotes that has profound regulatory effects on various metabolic and developmental processes (Close et al., 2010; Xing and Poirier, 2012). From a number of studies it became evident that lysine acetylation is involved in energy-homeostasis and regulation of carbon-flux, by directly altering NGE or the activities of key metabolic enzymes, such as glyceraldehyde 3-phosphate dehydrogenase and isocitrate dehydrogenase in *Salmonella enterica* (Wang et al., 2010), or mammalian mitochondrial acetyl-CoA-synthetase 2 (Hallows et al., 2006; Schwer et al., 2006). Furthermore, Kim et al. (2006) demonstrated that lysine acetylation patterns varied depending on nutritional status. Anderson and Hirschey (2012) estimated 35% of all mammalian mitochondrial proteins to have at least one acetylation site, and found pathways involved in the generation of energy, fatty acid metabolism, sugar metabolism, and amino acid metabolism to be significantly enriched in acetylated proteins. Important progress has also been made in identifying lysine-acetylated non-histone proteins in *Arabidopsis* (Finkemeier et al., 2011; Wu et al., 2011). These two studies describe a total number of 125 proteins to be lysine-acetylated in *Arabidopsis*, with cytochrome c representing the only mitochondrial protein. Only six common proteins were identified in both studies (Xing and Poirier, 2012), which suggests that the overall coverage was fairly low and that more extensive screens are likely to identify a much larger number of lysine-acetylated proteins in plants.

The huge potential of lysine acetylation acting as a metabolic sensor and regulator lies in the chemistry of the enzymes that catalyze this PTM. Lysine acetyltransferases (KATs) transfer the acetyl-moiety from acetyl-CoA to the target proteins. This suggests that the availability of the central metabolic intermediate acetyl-CoA or the acetyl-CoA/CoA ratio could be directly gaged by KATs (Xing and Poirier, 2012). As acetyl-CoA occurs in the cytosol and in organelles but cannot freely diffuse through membranes it has to be synthesized and metabolized independently in each compartment, which makes it a good indicator of local carbon status (Oliver et al., 2009). In *Arabidopsis* at least 12 KATs were identified by sequence homology and a number of them has been described to influence various developmental processes via acetylation of histones (Pandey et al., 2002; Earley et al., 2007; Servet et al., 2010). Information on KATs that acetylate non-histone proteins outside the nucleus is largely lacking. Only one mitochondrially localized KAT has been identified that acetylates lysine-residues of proteins belonging to the respiratory electron transport chain (ETC; Scott et al., 2012). It is well conceivable that there are other classes of KATs that remain to be discovered (Anderson and Hirschey, 2012).

In contrast, much more evidence is available for the reverse reaction catalyzed by lysine deacetylases (KDACs), in particular the KDAC class III of the so-called sirtuins (for “silent information regulation 2 homolog”). Sirtuins differ from the other three classes of deacetylases in their dependence on NAD^+^ as substrate and the release of *O*-acetyl-ribose and nicotinamide (NAM) as by-products of the deacetylation. The NAD^+^/NADH ratio reflects the energy status of a cell and several studies found that starvation conditions activate sirtuins in yeast and mammals, leading to transcriptional and metabolic adaptation via the deacetylation of histones, TFs, and metabolic enzymes (Zhong and Mostoslavsky, 2011; Houtkooper et al., 2012). These observations led to the hypothesis that protein acetylation could serve as a central switch between oxidative and fermentative metabolism, with acetyl-CoA, NAD^+^, KATs, and NAD^+^-dependent KDACs as key players (Guarente, 2011; Xing and Poirier, 2012). Although direct evidence is missing, such a model would allow the integration of MRR (Verdin et al., 2010). For example the activity of PGC1α (see above) is modulated by reversible acetylation through the KAT GCN5 and sirtuin 1 (SIRT1), depending on cellular acetyl-CoA and NAD^+^ status (Canto and Auwerx, 2009). Furthermore, SIRT3 is regarded to be the major mitochondrial deacetylase in mammals with many targets involved in mitochondrial metabolism. This includes central enzymes as for example complex I, II, and III of the ETC, isocitrate dehydrogenases and glutamate dehydrogenase in the TCA-cycle, mitochondrial acetyl-CoA-synthetase, and long-chain acyl-CoA dehydrogenase, which is involved in fatty acid oxidation (Anderson and Hirschey, 2012; Houtkooper et al., 2012). From these observations a common model has emerged suggesting that under starvation conditions SIRT3 is activated by increased NAD^+^ levels, stimulating oxidative metabolism and ATP-production (Zhong and Mostoslavsky, 2011). Changes in the cytosolic AMP/ATP ratio could be perceived by the AMP-activated protein kinase (AMPK, a homolog of SnRK1 mentioned above), which promotes catabolism and inactivates energy-consuming pathways (Verdin et al., 2010). Although the AMPK plant homolog SnRK1 is not allosterically regulated by the AMP/ATP ratio (Ghillebert et al., 2011) it is intimately linked with cellular carbon status. Consequently, a sirtuin-dependent metabolic regulatory pathway seems possible in plants. The *Arabidopsis* genome encodes for two sirtuin genes, of which one (AtSRT2) comes in seven different splice-forms. However, the subcellular localization and the possible functions in metabolic regulation remain to be determined. Apart from sirtuins *Arabidopsis* expresses at least 16 KDACs of other classes, the functions of which have just started to be unraveled (Pandey et al., 2002; Hollender and Liu, 2008). While some of them were confirmed to deacetylate histones in the nucleus to regulate developmental processes (e.g., HDA6; Wu et al., 2008; Yu et al., 2011), others, like HDA5, HDA8, and HDA14, were found to be expressed in the cytosol, chloroplasts, or mitochondria and seem to be involved in the acetylation of non-histone proteins, as for example α-tubulin in the case of HDA14 (Alinsug et al., 2012; Tran et al., 2012). The dependence on central metabolites as co-substrates and the potential to directly and reversibly influence NGE and enzymatic activities makes lysine acetylation a promising candidate for regulating plant metabolism on multiple levels including MRR.

## PTMs CONNECTED TO MITOCHONDRIAL REDOX STATE

Reactive oxygen species (ROS) are continuously produced as by-products of respiratory metabolism. Their production is increased under conditions of high matrix NADH/NAD^+^ ratios or when the ubiquinone pool is highly reduced in conjunction with a high membrane potential (Møller, 2001; Murphy, 2009). Hence, the mitochondrial redox state is intimately linked to mitochondrial energy metabolism, and is defined by the balance of ROS produced by metabolic processes and the availability of NADPH as reducing equivalent to sustain the antioxidant system. The mitochondrial antioxidant defense system has a key role in the detoxification of peroxides and thus is a prime target to affect redox signaling. PTMs on plant mitochondrial antioxidant enzymes have not yet been studied in detail, but phosphorylation sites of antioxidant enzymes have been identified in several studies and are awaiting further functional characterization (Nakagami et al., 2010).

Oxidative PTMs of proteins that arise from increased ROS production in mitochondria can either be irreversible or reversible. The chemistry of oxidative modifications on proteins is quite complex and their full potential is not explored yet. The best-characterized irreversible protein oxidation in mitochondria is carbonylation, which is regarded as an indicator of oxidative damage to the cell (Møller et al., 2011). The iron-sulfur cluster of the TCA-cycle enzyme aconitase for example is particularly prone to oxidative inactivation, which might activate MRR in plants by increasing mitochondrial citrate levels (Gray et al., 2004; Rhoads and Subbaiah, 2007). Reversible oxidative modifications only occur at cysteine and methionine residues of proteins, which can be reversed by sulfiredoxins (in case of cysteine sulfinic acids), thioredoxins (TRXs; in case of disulfides), glutaredoxins (GRXs; in case of disulfides and glutathione-mixed disulfides) and methionine sulfoxide reductases (in case of methionine sulfoxides). Disulfides formed during cysteine oxidation play important roles in regulating enzyme activities and protein functions in diverse metabolic processes as well as in transcriptional regulation and signaling in plants and animals (Foyer and Noctor, 2009; Konig et al., 2012; Murphy, 2012). In the matrix of plant mitochondria 50 TRX-linked proteins and 18 GRX-linked proteins have been identified from various metabolic processes (Balmer et al., 2004; Rouhier et al., 2005). However, the *in vivo* confirmation of the functional regulation of plant mitochondrial metabolism by TRXs and GRXs is still lacking. The plant mitochondrial disulphide proteome was further assessed in a study using diagonal gel electrophoresis (Winger et al., 2007), identifying 21 proteins from major metabolic pathways which form either inter- or intramolecular disulfides under oxidizing conditions. Catalytic cysteines are important for the activities of many enzymes involved in metabolism (e.g., cysteine proteases, ubiquitin ligases, and peroxidases) and signaling (e.g., tyrosine phosphatases). *S*-Glutathionylation is a reversible PTM of cysteine, which is regarded as mechanism to protect redox-active cysteines from irreversible inactivation beside its role in redox signaling (Gallogly and Mieyal, 2007). Both glycine decarboxylase, a key enzyme in the photorespiratory pathway, as well as galactonolactone dehydrogenase, a major enzyme in ascorbate synthesis, were inactivated by glutathionylation in plant mitochondria (Leferink et al., 2009; Palmieri et al., 2010). Hence, glutathionylation could play a role in the temporary protection of these metabolic enzymes under conditions of oxidative stress. Metabolic changes resulting from inhibitions of these enzymes could then play a role in MRR. Irreversibly inactivated proteins can be degraded in all mitochondrial subcompartments (Fischer et al., 2012). Recent work in *C. elegans* demonstrated that the mitochondrial peptide exporter HAF-1 is required for MRR (Haynes et al., 2010). Møller and Sweetlove (2010) suggested that oxidatively modified peptides in particular could convey specific information to regulate oxidative stress-induced MRR in plants. Novel proteomic approaches using bifunctional thiol-specific alkylation reagents coupled to an epitope tag, or to a fluorescent or isotope-label will provide useful tools to further explore the functional significance of the mitochondrial redox proteome and its putative role in regulating MRR in plants (Held and Gibson, 2012).

## FUTURE DIRECTIONS

A recent in-depth bioinformatics analysis estimated the plant mitochondrial proteome to contain about 2500 proteins (Cui et al., 2011). Many of these proteins will contain multiple PTMs of different types, which will consequently alter protein functions or even localization of proteins within the mitochondria. When thinking of the role of PTMs in signaling, primarily the classical phosphorylation-based MAPK-cascades come to mind, which are often initiated by receptor kinases. However, it is also conceivable that different types of PTMs could be series-connected or could interact in a codified crosstalk. A recent *in silico* study demonstrated that lysine acetylation sites have a great potential to affect nearby phosphorylation, methylation, and ubiquitination sites (Lu et al., 2011). Such a crosstalk was indeed observed in an* in vivo* study using the genome-reduced bacterium *Mycoplasma pneumonia*e (van Noort et al., 2012). Deletion of the only two protein kinases and a unique protein phosphatase modulated lysine acetylation patterns, while in return deletion of the only two KATs had an impact on protein phosphorylation. Hence, many scenarios are conceivable that include phosphorylation-based signaling cascades regulated by various types of PTMs. Just as conceivable is the inverse scenario, having, phosphorylation or lysine acetylation regulating the activity of metabolic enzymes which would affect metabolic signaling. Such a complex PTM-based crosstalk is for example demonstrated in the regulation of the activity of the mitochondrial manganese superoxide dismutase (MnSOD). MnSOD is responsible for the decomposition of superoxide to hydrogen peroxide in the mitochondrial matrix. The activity of MnSOD can be modified by several PTMs including phosphorylation, lysine acetylation, and tyrosine nitration in mammals (Yamakura et al., 1998; Hopper et al., 2006; Ozden et al., 2011), while phosphorylation, carbonylation, and oxidative degradation of MnSOD have been observed in plants (Sweetlove et al., 2002; Kristensen et al., 2004; Juszczuk et al., 2007). Removal of phosphorylation and lysine acetylation from MnSOD were both shown to increase its activity, however, these occur under different physiological conditions (Finley and Haigis, 2009; Foster et al., 2009; Fukai and Ushio-Fukai, 2011; Ozden et al., 2011). Hence both modifications are probably connected in a codified crosstalk and will impact on mitochondrial redox signaling.

Several key questions will need to be answered in future research to fully understand the complexity and regulation of the mitochondrial proteome in dependence on environmental or metabolic cues: (1) Do different PTMs identified on the same proteins occur at the same time and if not what is their sequential arrangement?, (2) What is the physiological role of the different PTMs with regard to protein function?, and (3) Do different PTMs influence each other? To investigate these questions in detail and to further explore the role of PTMs in MRR, sophisticated proteomic analyses will be necessary that allow a time-resolved and in-depth inventory of the mitochondrial proteome and its modifications under conditions that trigger MRR. Furthermore, shot-gun proteomics approaches that analyze fractionated whole cell proteomes rather than sub-proteomes from isolated organelles might be more suitable for such type of analysis. Mitochondrial isolation procedures often take several hours and can have broad impacts on mitochondrial PTMs such as oxidative modifications. Several examples of PTM-based regulation of central mitochondrial proteins in animals demonstrate the need for further dissection of the functions of PTMs in plant mitochondrial signaling. Furthermore, autotrophic plant tissues differ fundamentally from animal heterotrophic tissues in terms of energy production, consumption, and homeostasis, and it is most likely that novel plant-specific regulatory mechanisms will be identified.

## Conflict of Interest Statement

The authors declare that the research was conducted in the absence of any commercial or financial relationships that could be construed as a potential conflict of interest.
